# Exposure to Heatwaves and High Depressive Symptoms among Older Adults in Mexico

**DOI:** 10.1093/geroni/igaf122.4322

**Published:** 2025-12-31

**Authors:** Brian Downer, Joseph Saenz, Rebeca Wong, Emma Aguila, Jorge Peniche

**Affiliations:** University of Texas Medical Branch, Galveston, Texas, United States; Arizona State University, Phoenix, Arizona, United States; University of Texas Health San Antonio, San Antonio, Texas, United States; University of Southern California, Los Angeles, California, United States; University of Southern California, Los Angeles, California, United States

## Abstract

Heatwaves can increase the risk of depressive symptoms among older adults. However, it is unknown whether this association is modified by the structural and physical characteristics of a person’s home. We used data from 8,952 participants in the Mexican Health and Aging Study who were aged 60 and older in 2018. Participants were linked to municipality-level data for daily maximum temperatures from May 1st to August 31st of each year from 1981-2018. A heatwave was defined as > 2 consecutive days with a maximum daily temperature >95th percentile of municipality-specific temperatures from 1981-2017. We created a variable for the total number of heatwaves (0, 1, >2) in summer 2018. Latent class analysis was used to define home types based on the materials for the roof, walls, floor, and the number of levels and rooms. Logistic regression models that adjusted for demographic characteristics were used. Participants who experienced >2 heatwaves had 1.15 (95% CI = 1.01-1.31) higher odds for high depressive symptoms compared to those who experienced no heatwaves. The interaction term was not significant, but this association was significant for participants living in a single-story home with mixed floor materials, concrete roof, and some rooms (OR = 1.27, 95% CI = 1.05-1.55), but not in a multi-story home with wood floors, concreate roof, and many rooms (OR = 1.16, 95% CI = 0.91-1.48) or a single-story home with concrete floors, laminate roof, and a few rooms (OR = 0.92, 95% CI = 0.71-1.19). Heatwaves are a risk factor for high depressive symptoms, the effects of which may differ based on a home’s characteristics.

